# Automatic quantification of REM sleep without atonia reliably identifies patients with REM sleep behavior disorder: a possible screening tool?

**DOI:** 10.1007/s10072-024-07532-6

**Published:** 2024-05-22

**Authors:** Raffaele Mancini, Pietro Mattioli, Francesco Famà, Laura Giorgetti, Francesco Calizzano, Miki Nikolic, Rune Frandsen, Poul Jennum, Silvia Morbelli, Matteo Pardini, Dario Arnaldi

**Affiliations:** 1 Department of Neuroscience, Rehabilitation, Ophthalmology, Genetics, Maternal and Child Health (DINOGMI), University of Genoa, Genoa, Italy; 2 Neurofisiopatologia, IRCCS Ospedale Policlinico San Martino, Genoa, Italy; 3 Clinica Neurologica, IRCCS Ospedale Policlinico San Martino, Genoa, Italy; 4 Danish Center for Sleep Medicine, Department of Clinical Neurophysiology, Rigshospitalet, Copenhagen, Denmark; 5 Nuclear Medicine Unit, AOU Città della Salute e della Scienza di Torino, Turin, Italy; 6 Department of Medical Sciences, University of Turin, Turin, Italy

**Keywords:** RBD, DAT-SPECT, PE2IPET, RWA

## Abstract

**Background:**

REM Sleep Behavior Disorder (RBD) is characterized by absence of physiological muscle atonia during REM sleep (REM sleep without atonia, RWA). Nigro-striatal dopaminergic impairment is a feature of Parkinson disease (PD) and can be identified in prodromal stages as well, such as idiopathic RBD (iRBD). Aims of this study are to explore the efficacy of an automatic RWA quantification in identifying RBD patients and the correlation between RWA and nigro-striatal dopaminergic function.

**Methods:**

Forty-five iRBD, 46 PD with RBD, 24 PD without RBD patients and 11 healthy controls were enrolled in the Genoa Center (group A) and 25 patients with iRBD (group B) were enrolled in the Danish Center. Group A underwent brain [123I]FP-CIT-SPECT and group B underwent brain [18F]PE2I-PET as measures of nigro-striatal dopaminergic function. Chin muscle activity was recorded in all subjects and analyzed by applying a published automatic algorithm. Correlations between RWA and nigro-striatal dopaminergic function were explored.

**Results:**

The automatic quantification of RWA significantly differentiated RBD from non-RBD subjects (AUC = 0.86), although with lower accuracy compared with conventional visual scoring (AUC = 0.99). No significant correlation was found between RWA and nigro-striatal dopaminergic function.

**Conclusion:**

The automatic quantification of RWA is a reliable tool to identify subjects with RBD and may be used as a first-line screening tool, but without correlations with nigro-striatal dopaminergic functioning.

## Introduction

REM sleep behavior disorder (RBD) is a REM sleep parasomnia characterized by sleep related complex motor behaviors with dream enactment [1]. This condition can be suspected by anamnestic reports, but the diagnosis requires a polysomnographic (PSG) confirmation of enhanced muscle activity during REM sleep (REM sleep without atonia, RWA) [2]. To date, the PSG visual analysis remains the gold standard to efficiently document RWA [3, 4]. However, this method is time consuming and requires a skilled and trained staff [4–6]. For this reason, automated and semi-automated methods to identify RWA on PSG recordings have been developed [7–9]. Recently, an algorithm for the automatic quantification of RWA was developed by the Danish Center for Sleep Medicine, showing performances comparable to other automated methods, with good reproducibility and ease of measurements in a clinical setting [10]. However, it was not validated in an independent sample of patients. Therefore, the first aim of the present study is to validate the Danish automatic RWA quantification method in an independent cohort of subjects.

Idiopathic RBD (iRBD) is widely recognized as a prodromal stage of α-synucleinopathies, such as Parkinson’s Disease (PD), Dementia with Lewy bodies (DLB) and Multiple Systems Atrophy. The vast majority of the subjects affected by iRBD will develop overt α-synucleinopathies during their lifetimes [11, 12]. Neurodegeneration of the nigro-striatal dopaminergic pathway is a distinctive feature of most α-synucleinopathies (mainly PD and DLB) and can be identified in a significant portion of iRBD patients, even before the development of overt motor signs [13]. The presence of an altered nigro-striatal pathway represents a prognostic marker of short-to-medium term phenoconversion to overt neurodegenerative disease in iRBD patients [13]. Recently, RWA has been described as a potential prognostic biomarker of disease progression, associated with earlier phenoconversion in iRBD [14]. Nigro-striatal dopamine pathway alteration and RWA depend on different pathological mechanisms, i.e. degeneration of the dopaminergic neurons in substantia nigra pars compacta and degeneration of locus coeruleus/subcoeruleus complex respectively [15]. However, it has been suggested that they both express alpha-synuclein-related neurodegeneration, even if at different levels. Moreover, a recent study showed a significant association between RWA and nigro-striatal dopaminergic function, as measured by [123I]FP-CIT-SPECT, in a group of iRBD patients [16]. Thus, the second aim of the present study was to investigate the association between both automatic and visual RWA scoring and nigro-striatal dopaminergic function, as investigated with the established [123I]FP-CIT-SPECT, and with a new technique, [18F]PE2I-PET, in a group of subjects with RBD, both in the ‘idiopathic’ form and associated with overt PD, in comparison with subjects without RBD (healthy controls and PD patients without RBD).

## Materials and methods

### Study design

A two-centers retrospective study with data collected from 2008 to 2020, consisting of two aims:

1. Validation of an algorithm for the automatic computation of RWA scores developed by the Danish Center for Sleep Medicine in an independent group of subjects from the Genoa Sleep Center (group A). The group included iRBD patients, PD patients and healthy controls. We then evaluated the correlation between the results from the RWA analysis and the nigro-striatal dopaminergic function quantified with 123I-Ioflupane Single Photon Emission Computed Tomography [123I]FP-CIT-SPECT (DAT SPECT);
2. Correlation between the results from the automated RWA analysis and the nigro-striatal dopaminergic function quantified with N-(3-iodopro-2E-enyl)-2β-carbomethoxy-3β-(4'-methylphenyl) nortropane positron emission tomography ([18F]PE2I-PET) in a group of iRBD patients from the Danish Center for Sleep Medicine (group B).

### Participants

As for aim 1, we enrolled 126 subjects from the Genoa Sleep Center (IRCCS Ospedale Policlino San Martino): 45 patients with iRBD, 24 PD patients without RBD, 46 PD patients with RBD and 11 healthy controls (HC). As for HC patients, they were selected from subjects that performed [123I]FP-CIT-SPECT DAT-SCAN at our nuclear medicine unit following good clinical practice (GCP) (e.g. for the differential diagnosis of PD and essential tremor or iatrogenic parkinsonism) and had normal nigro-striatal pathway.

As for aim 2, we enrolled 25 iRBD patients from Danish Center for Sleep Medicine (Rigshospitalet).

The following inclusion criteria were defined: iRBD diagnosis was video-polysomnography (vPSG) proven; PD diagnosis was made in accordance with international diagnostic criteria [17]. All PD patients were de novo and drug naïve. RBD in PD patients was confirmed with PSG in accordance to the ICSD-3 criteria [18]. Exclusion criteria were the presence of any other neurological or psychiatric clinically relevant condition including stroke, major traumatic brain injury, brain tumor, epilepsy, presence of clinically relevant pulmonary, gastrointestinal, renal, hepatic, endocrine or cardiovascular disease. Moreover, all the subjects underwent a complete neuropsychological and daily living functioning evaluation to rule out the presence of Dementia. The movement disorder society unified Parkinson disease rating scale, motor section (MDS-UPDRS-III) was administered to the iRBD and HC subjects to rule out parkinsonism.

### PSG manual and automatic RWA analysis

All subjects, including iRBD patients, PD patients and healthy controls, underwent a single night PSG recording performed by expert technicians. Before the PSG recording, we ascertained that all the subjects were free from neurotropic drugs for at least two weeks. PSG monitoring was done during a single night session according to the international American Association of Sleep Medicine (AASM) criteria [5]. For the EEG recording we used a six derivations montage: F3-A2, F4-A1, C3-A2, C4-A1, O1-A2, O2-A1 (“10–20” International System). We used two electrooculography (EOG) derivations, one for the right eye and one for the left one, with contralateral mastoid reference. Electromyography (EMG) was recorded with three surface derivations: submentalis muscle and bilateral tibialis anterior. Moreover, the following supplemental derivations were used: thoracic and abdominal bands for respiratory effort; a nasal cannula to evaluate the oronasal airflow and determine the presence of potential sleep apnea/hypopnea; an oxygen saturation and a single electrocardiographic (ECG) derivation. The apnea/hypopnea index (AHI) and periodic leg movements index (PLMI) PSG indices were also acquired and used for statistical analyses.

Quantification of submentalis muscle activity was obtained during the following sleep stages: REM sleep, S1, S2, S3, NREM (S1 + S2 + S3). In all stages, the following parameters were obtained: percentage of mini epochs (% mini epochs); duration of submentalis muscle movements during each stage (duration); percentage of submentalis muscle movements during each stage (% duration).

To quantify RWA on EMG recording we used both the visual method and the automatic algorithm developed by the Danish Center for Sleep Medicine [10]. For the visual scoring, we used the ‘tonic’, ‘phasic’ and ‘any’ RWA parameters on 30-s epochs according to Frauscher et al. [3]. Visual RWA scoring was performed by an experienced neurologist blinded from clinical and imaging parameters.

Briefly, the automatic RWA score algorithm is based on an amplitude curve (AC) generated within a sliding 51-sample window progressing though the EMG signal with definite duration, motor activity detection threshold (MADT) and inter-event interval (IEI). From now on we will refer to this method as ‘Automatic RWA’. For this method, to identify a motor activity event (MAE) we used the following factors that resulted in the best performances in differentiating RBD from HC when applied:

- Baseline analyzed with a moving window.
- MADT four times baseline.
- Event Duration 0.3 s.
- IEI 0.5 s.
- Exclusion of no events.
- REM sleep total percentage and 3-s mini-epochs.

The algorithm produces an ‘Automatic RWA’ parameter with values spanning from 0 to 1. This parameter stands for the number of 3-s mini epochs in which the MAE occupy more than 50% of the mini epoch among the total amount of REM 3 s mini-epochs. The value 0 indicates the presence of complete muscle atonia, whereas the value 1 indicates complete absence of muscle atonia.

### 123I-Ioflupane Single Photon Emission Computed Tomography ([123I]FP-CIT-SPECT)

PSG confirmed iRBD patients and PD patients from the Genoa Center underwent [123I]FP-CIT-SPECT to evaluate nigro-striatal dopaminergic functioning. [123I]FP-CIT-SPECT data were all obtained in the same laboratory and gamma-chamber, a two-headed Millennium VG camera (GE Healthcare, 3000 N Grandview Blv Waukasha, WI, US, 53,188). Images were acquired after intravenous administration of [123I]FP-CIT (DaTSCAN, GE Healthcare) according to the European Association of Nuclear Medicine (EANM) guidelines [19]. By means of an electronic zoom (zoom factor = 1.8) applied during the data collection phase the pixel size of the acquisition matrix was 2.4 mm. Isotropic voxels with 2.33-mm sides were sampled with a second digital zoom applied in the reconstruction phase. Ordered Subsets Expectation Maximation (OSEM) algorithm with a series of subsequent filters (3D Gaussian filter with Full Width-Half Maximum, FWHM = 8 mm) were also used in the reconstruction phase. BasGan software version 2 [20] based on a high-definition, three dimensional striatal template, derived from Talairach’s and Tournoux atlas was used to process the reconstructed [123I]FP-CIT-SPECT images. Putamen and caudate uptake levels were normalized using the occipital lobes uptake as the background reference region to compute bilateral specific to non-displaceable binding ratios (SBRs) with the formula: (caudate or putamen uptake – background uptake)/background uptake). SBR were converted in Z score (Z caudate and Z putamen) normalized by age, using a database containing more than a hundred normal subjects from European ENC-DAT database [20]. Mean SBR and Z values of the two caudate and of the two putamen were used in statistical analysis [21].

### N-(3-iodopro-2E-enyl)-2β-carbomethoxy-3β-(4'-methylphenyl) nortropane (PE2I) PET

**Fig. 1:**
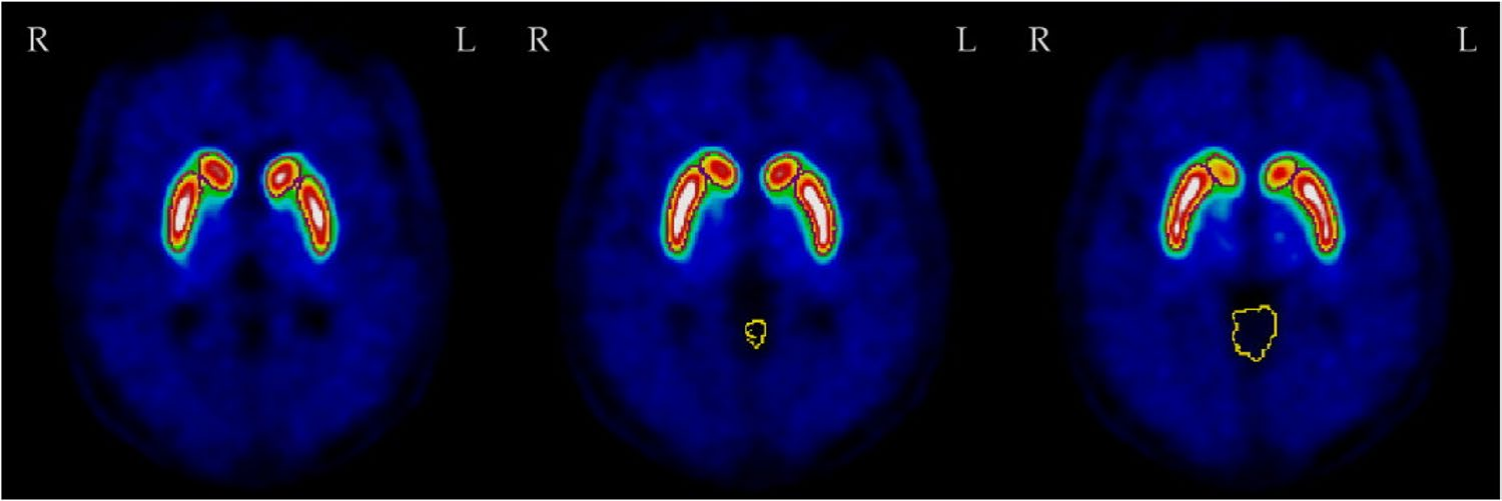
Normal [18F]PE2I-PET of patient 7, three consecutive slices of basal ganglia

**Fig. 2:**
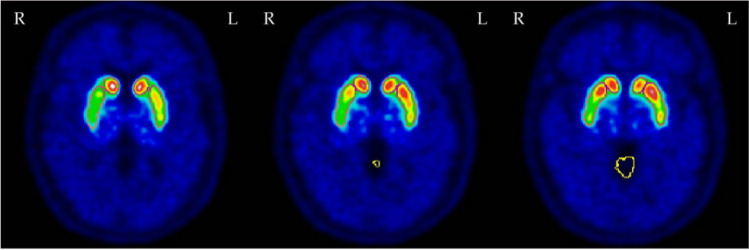
Abnormal [18F]PE2I-PET of Patient 17, three consecutive slices of basal ganglia

PSG confirmed iRBD patients and PD patients from Rigshospitalet underwent [18F]PE2I-PET to evaluate nigro-striatal dopaminergic functioning. [18F]PE2I-PET was a 10 min scan, commenced 30 min after tracer administration (mean activity: 204 ± 12 MBq, range: 180 255 MBq) on a Biograph mCT (Siemens Healthineers, Erlangen, Germany). The axial FOV is 22 cm with a resolution FWHM 1 cm from the center of 4.4 for both axial and transverse direction. A low dose CR scan was used for attenuation correction (voxel size 0.59 mm 2, 512 × 512 matrix size, 120 KVP). All PET scans were preceded by a low-dose CT for attenuation correction and a diagnostic CT-scan. [18F]PE2I-PET images have been standardized to Z scores (Z caudate and Z putamen) as previously described [22]. This protocol for PE2I imaging is currently the standart clinical scan used for diagnosing Parkinson’s disease at Rigshospitalet. Mean SBR and Z values of the two caudate and of the two putamen were used in statistical analysis. Figures 1 and 2 show, respectively, a normal and an abnormal [18F]-PE2I-PET scan.

### Statistical analysis

In a first descriptive analysis we compared clinical, demographic, and polysomnographic data between the four study groups from group A: HC, iRBD, PD without RBD (PDnoRBD), PD with RBD (PD + RBD).

Continuous variables were compared using univariate variance (ANOVA) and Bonferroni post-hoc analysis. Categorical variables were compared using Fisher’s exact tests.

Automatic RWA and visual scoring of motor activity were compared using univariate analysis of variance (ANOVA).

To investigate the ability of the visual RWA parameters (‘tonic’, ‘phasic’ and ‘any’) and the Automatic RWA to distinguish between subjects without RBD (HC and PDnoRBD) and subjects with RBD (iRBD and PD + RBD), a receiver operating characteristic (ROC) was used. Subsequently, ROC curves of the four RWA parameters were compared to explore whether one of the four parameters showed better performances in distinguishing subjects with RBD from subjects without RBD. Finally, Pearson correlation was also used to explore possible correlations between RWA parameters, both visual and automatic, and nigro-striatal dopaminergic functioning quantified with [123I]FP-CIT-SPECT.

In the second part of the study, regression analysis was used to investigate the correlation between the quantification of motor activity during REM and NREM sleep (S1, S2 ed S3) using the automatic method and nigro-striatal dopaminergic functioning quantified with [18F]PE2I-PET. Sleep motor activity values were used as independent variable whereas [18F]PE2I-PET imaging values were used as dependent variables. Coefficients of determination, i.e. R square, were computed to verify the prediction strength of the linear regression model. F values were used as a mean of determining statistically significancy, expressing the relevance of the independent variable.

A *p* value < 0.05 was considered as statistically significant. Statistical analysis was performed using STATA software. Bonferroni correction for multiple comparisons was applied.

## Results

### Genoa sleep center subjects

#### Clinical and polysomnographic profiles

The main clinical, demographic, and polysomnographic characteristics are summarized in Table 1.

**Table 1:** Main clinical, demographic and polysomnographic characteristics of subjects with iRBD, PD with RBD (PD + RBD), PD without RBD (PDnoRBD) and healthy controls (HC), reported as mean values

	HC (*n* = 11)	PDnoRBD (*n* = 24)	iRBD (*n* = 45)	PD + RBD (*n* = 46)	*P* value
Sex, M	5 (45.45%)	13 (54.16%)	36 (80.00%)	29 (63.04%)	0.028
Age (years)	67.27 ± 11.41	66.67 ± 9.47	68.51 ± 7.79	72.46 ± 6.25	0.015
Phasic RWA	3.28 ± 3.41	5.77 ± 3.94	45.38 ± 23.03	29.77 ± 20.22	< 0.0001
Tonic RWA	1.07 ± 1.74	2.73 ± 2.59	38.35 ± 25.40	45.44 ± 29.97	< 0.0001
Any RWA	4.90 ± 4.77	7.78 ± 4.92	59.61 ± 23.48	57.35 ± 24.81	< 0.0001
Automatic RWA	0.13 ± 0.20	0.11 ± 0.07	0.28 ± 0.18	0.39 ± 0.21	< 0.0001
AHI	4.87 ± 6.80	12.84 ± 18.61	5.72 ± 7.84	16.41 ± 18.12	NS
PLMI	20.61 ± 40.86	5.75 ± 9.94	25.22 ± 37.84	24.24 ± 27.55	NS
Caudate, SBR	-	2.55 ± 1.01	3.52 ± 1.10	2.24 ± 0.62	< 0.0001
Putamen, SBR	-	1.53 ± 0.65	2.85 ± 0.95	1.36 ± 0.51	< 0.0001
Caudate, z scores	-	-1.69 ± 0.33	-1.24 ± 0.42	-1.69 ± 0.30	< 0.0001
Putamen, z scores	-	-1.92 ± 0.36	-1.29 ± 0.28	-1.85 ± 0.30	< 0.0001

AHI (apnea/hypopnea index), *NS* not significant, *PLMI* (periodic leg movement index), *SBR* specific to non-displaceable binding ratios.

RWA quantification parameters were significantly different among the four groups (iRBD, PD + RBD, PDnoRBD and HC, *p* < 0.0001). Post-hoc Bonferroni analysis shows that HC and PDnoRBD subjects had no statistically significant differences between them, therefore we considered them as a single group (Subjects without RBD). Similarly, iRBD and PD + RBD subjects had no statistically significant differences between them, therefore we considered them as another single group (Subjects with RBD).

As expected, Automatic RWA showed a statistically significant positive correlation with visual RWA parameters, that is phasic RWA (R = 0.57, *p* < 0.0001), tonic RWA (R = 0.53, *p* < 0.0001) and any RWA (R = 0.62, *p* < 0.0001).

#### Automatic algorithm analysis and [123I]FP-CIT-SPECT nigro-striatal dopaminergic function correlations

**Fig. 3:**
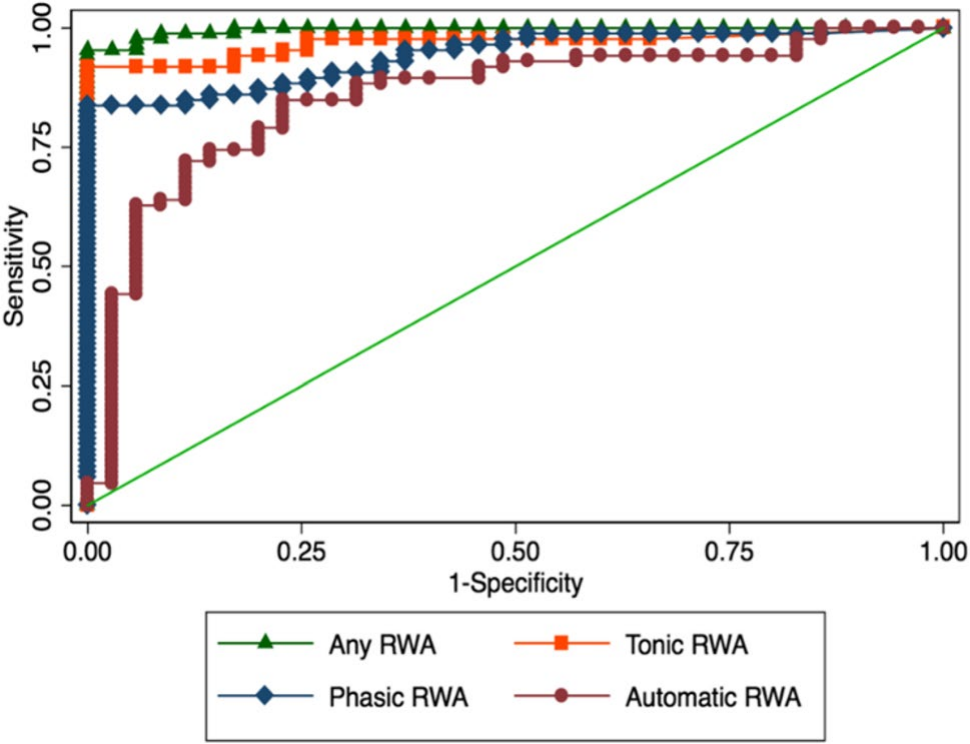
ROC curves distribution for the four RWA parameters considered

ROC curves analysis of subjects with RBD and subjects without RBD are shown in Fig. 3 and summarized in Table 2. Table 2 also shows the comparison between the AUC of the tonic, phasic and Automatic RWA parameters and the Any RWA parameter, which was the one that yielded the higher AUC and was therefore used as a gold standard.

**Table 2:** ROC analysis of RWA parameters. Comparison between subjects with RBD (iRBD and PD + RBD) e subjects without RBD (HC and PDnoRBD)

	AUC (CI 95%)	Standard Error	Cut point	Specificity	Sensitivity	AUC vs Any RWA (p value)
Any RWA	0.99 (0.99–1.00)	0.003	18%	1.00	0.96	n.a
Tonic RWA	0.97 (0.94–0.99)	0.015	9%	1.00	0.91	0.189
Phasic RWA	0.95 (0.91–0.98)	0.019	12%	1.00	0.85	0.011
Automatic RWA	0.86 (0.78–0.93)	0.039	0.15	0.77	0.85	< 0.001

Compared with Any RWA, tonic RWA parameter did not show any statistically significant difference in differentiating subjects with RBD from subjects without RBD. Conversely, phasic RWA and the Automatic RWA parameters resulted as significantly inferior to Any RWA in differentiating subjects with RBD from subjects without RBD.

**Fig. 4:**
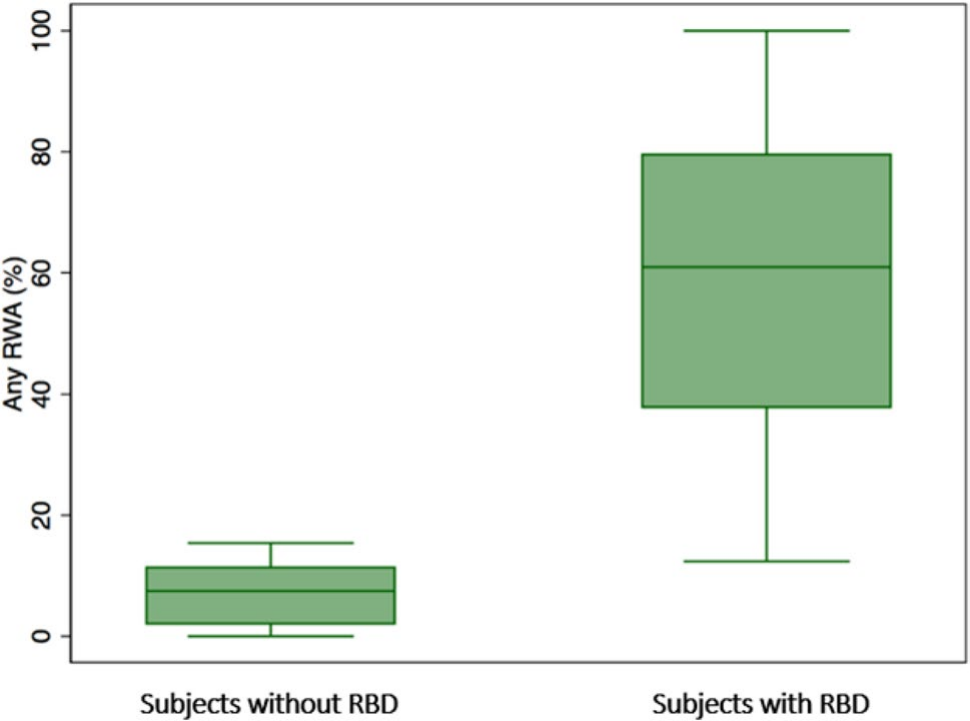
Box plot of Any RWA values, in subjects without RBD (HC e PDnoRBD) and in subjects with RBD (iRBD e PD + RBD)

**Fig. 5:**
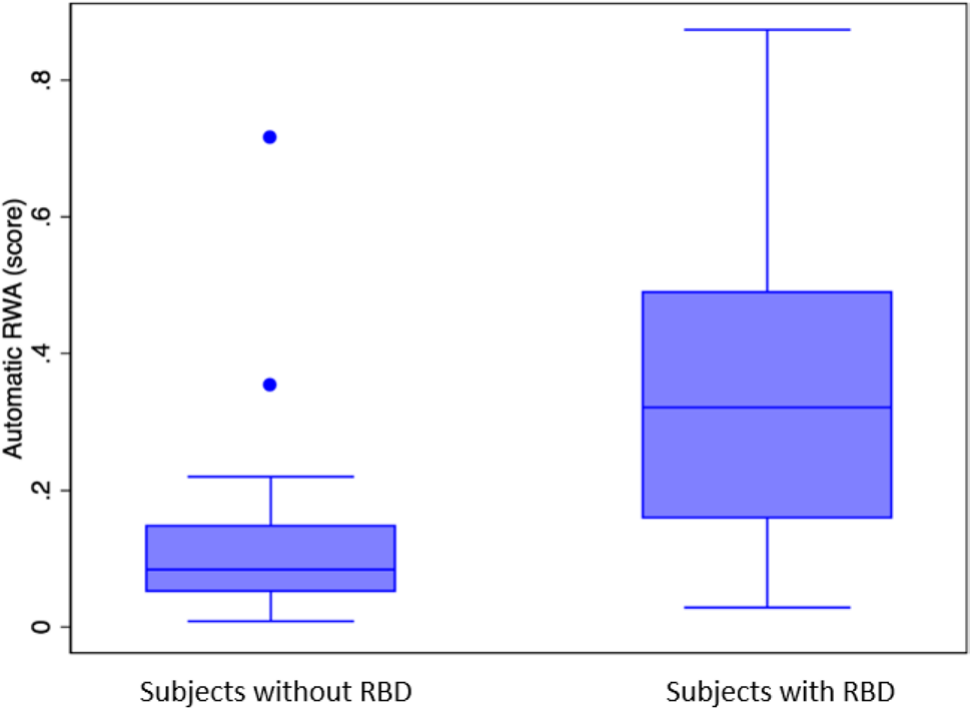
Box plot of Automatic RWA values, in subjects without RBD (HC e PDnoRBD) and in subjects with RBD (iRBD e PD + RBD)

Any RWA and Automatic RWA values of the two groups (subjects with RBD and subjects without RBD) are shown in Figs. 4 and 5 respectively.

A correlation analysis between the two PSG index AHI (apnea/hypopnea index) and PLMI (periodic leg movement index) and REM muscle activity was made. AHI did not show any significant correlation with REM motor activity, whereas PLMI index showed a significant correlation with Automatic RWA parameter (R = 0.4261, *p* = 0.0001) and, albeit weak, with Any RWA (R = 0.2802, *p* = 0.0476).

No statistically significant correlation was found between the [123I]FP-CIT-SPECT values, both as mean values and Z-score, and the four RWA parameters.

### Danish sleep center subjects

#### Automatic algorithm analysis and [18F]PE2I-PET nigro-striatal dopaminergic function correlations

The second part of the study involved 25 iRBD patients (65 ± 8.68 years, 17 men) from the Danish Sleep Center. Automatic RWA was 0.27 ± 0.19.

The main polysomnographic and [18F]PE2I-PET values are summarized in Table 3.

**Table 3:** iRBD with [18F]PE2I-PET scan

	iRBD (*n* = 25)
Sex, M	17 (68%)
Age, years	65 ± 8.68
Striatum Right z-score	-1.36 ± 1.11
Striatum Left z-score	-1.43 ± 1.15
Striatum Right SBR	3.24 ± 0.88
Striatum Left SBR	3.23 ± 0.89
Putamen Right z-score	-1.48 ± 1.23
Putamen Left z-score	-1.54 ± 1.23
Putamen Right SBR	3.24 ± 0.94
Putamen Left SBR	3.22 ± 0.95
Ratio Putamen/Caudatus Right SBR	1 ± 0.11
Ratio Putamen/Caudatus Left SBR	1 ± 0.1
Automatic RWA	0.27 ± 0.19
% duration in REM	25.09 ± 19.67
% duration in S1	19.97 ± 12.73
% duration in S2	21.06 ± 16.5
% duration in S3	10.71 ± 15.91
% duration in NREM	21.08 ± 16.02

No significant correlation was found between Automatic RWA and [18F]PE2I-PET data.

## Discussion

In this study we validated an algorithm for the automatic quantification of RWA in two independent cohorts of patients with RBD. Moreover, we directly compared the RWA values, both obtained with the automatic and the visual methods, and nigro-striatal dopaminergic functions quantified with two different nuclear medicine tools ([123I]FP-CIT-SPECT and [18F]PE2I-PET). No significant correlation was found between RWA metrics and nigro-striatal dopaminergic function either measured with SPECT or PET dopamine transporter imaging.

In recent years, many different software for the automatic quantification of RWA have been developed [8, 9]. These methods showed a good capability to identify RBD with comparable performances. However, each software has different algorithm pipelines and parameters. For now, these novel approaches are used only in research settings, but they may represent an important resource in the diagnosis of RBD as a tool for a fast and standardized analysis of large sets of PSG data.

The first aim of this study was to validate an automatic algorithm for the quantification of muscle activity during REM sleep, developed by the Danish Center for Sleep Medicine, in an independent group of subjects with RBD and without RBD from the Genoa Sleep Center. The method was the one developed by Frandsen et al. [10] using the factors that yielded the best performances in differentiating RBD from non RBD subjects.

These factors resulted from a comparison between 648 different combinations for the analysis of more than a million motor events. This automatic method was the first one to compare different types of baselines, the best of which resulted to be the one using a moving window baseline. With this kind of approach, it is possible to adapt the baseline during the night and throughout the PSG recording. This could be useful especially with PD patients undergoing medical therapy, whose effect and its eventual wearing off on muscle activity must be considered when taking a PSG recording. Moreover, this model was the first one to systematically define different values of duration and separation of motor events.

In this study, four different RWA quantification methods were studied, three visual methods (any, tonic and phasic) and the automatic one, in an independent group of subjects affected by RBD, both in its idiopathic form and the PD related one.

The automatic method ‘Automatic RWA’ showed a good capability to differentiate subjects with RBD from subjects without RBD, with an AUC of 0.86, like the one previously reported (AUC = 0.81) [10]. However, the automatic method AUC was significantly inferior compared to the “Any RWA” in visual scoring, which was the single parameter that showed better performances in identifying RBD, with an AUC of 0.99 and a cut-point of 18%, along with the tonic RWA parameter. Thus, the results of this study confirmed the superiority of the visual method as well as its role as the gold standard in RWA identification [3]. To note, according to international criteria, RBD diagnosis can be made only when enhanced muscle tone at visual analysis is found. Therefore, it is not surprising that the visual ‘any’ RWA parameters achieved nearly perfect diagnostic ability [23]. Nevertheless, the automatic method may be used as a first screening tool identifying a cut-off with maximum sensibility to subsequently rule out the false positives with the visual method.

The Automatic RWA showed an AUC comparable to that of other already available automatic methods, such as that of Meyer et al. (AUC = 0.82) [7] and the REM Atonia Index (RA) (AUC = 0.83) [8]. The automatic method introduced by Kempfner et al. has shown the best AUC of 0.993 [9], but this method is technically complex and hardly feasible in a clinical setting, requiring five EMG derivations. Anyway, a direct comparison between these automatic methods is beyond the aims of this study.

Each RWA parameter resulted independent from AHI index. This finding is significant because snoring and sleep-related breathing episodes in patients with obstructive sleep apnea disorder (OSAS) often produce artifacts that can make a proper quantification of RWA more difficult, especially when only the submentalis muscle is used [24].

Conversely, Any RWA and Automatic RWA showed a correlation with the PLMI index, more evident for the Automatic RWA parameter. This seems to suggest that the automatic method, compared with the visual analysis, has a lower degree of accuracy in differentiate RWA motor activity from artifacts produced by limb movements in Periodic Limb Movement Disorder in sleep (PLMD) [25]. This could explain, at least in part, the lower accuracy of the automated method in distinguishing patients with RBD from those without this disorder when compared to the visual analysis.

The extent of excessive muscle activity in REM sleep quantified with the four different methods (automatic and visual) did not show any significant correlation with nigro-striatal dopaminergic function quantified with [123I]FP-CIT-SPECT in the Genoa group. Moreover, in the second part of the study, we explored the correlation of EMG muscle activity quantified with the Automatic RWA automated method during REM and NREM sleep with nigro-striatal dopaminergic function quantified by [18F]PE2I-PET in a group of iRBD patients, showing again no statistically significant correlation. Therefore, our results suggest that quantitative excessive motor activity during REM sleep in patients with RBD, both in its idiopathic form and its PD related one, seems to be independent from the magnitude of nigro-striatal dopaminergic deafferentation.

Some studies previously reported that nigro-striatal dopaminergic function quantified with [123I]FP-CIT-SPECT did not show statistically significant correlations either with age of onset and duration of RBD [26] or with EMG activity [27, 28]. In another study [29], the quantification of RWA with RAI also did not show any significant correlation when compared with nigro-striatal dopaminergic function quantified with [123I]FP-CIT-SPECT. However, in a recent study, a modest correlation was found between RWA metrics and DAT binding ratios [16]. The present study does not confirm this latter result. A possible explanation could be that, although neurodegeneration due to alpha-synucleinopathy causes both RBD and motor symptoms typical of PD, this occurs affecting different cerebral and brainstem structures. It is known that parkinsonism in PD is due to mesencephalic substantia nigra degeneration [30]. Conversely, RBD is thought to be due to degeneration of the coeruleus/subcoeruleus complex [15]. Indeed, this area has been shown to be altered in subjects with iRBD [31] and PD with RBD [32]. It is given that neurodegeneration in alpha-synucleinopathy patients starts in lower parts of the brainstem including primarily the vagus nuclei, the coeruleus/subcoeruleus complex being affected first and much later involving the dopaminergic nuclei [33]. This likely explains the findings in this study, that is the lack of correlation between RWA activity and the nigrostriatal dopaminergic functioning. Indeed, the pathophysiology of RWA includes a disfunction of noradrenergic pathways instead of dopaminergic pathways in iRBD, and a correlation between noradrenergic activity and RBD has been described in PD patients [34, 35]. Supporting this hypothesis, the extent of motor activity during REM sleep was significantly correlated with the extent of damage of the coeruleus/subcoeruleus complex [34, 35]. Finally, nigro-striatal pathway alterations are highly heterogeneous in iRBD patients, spanning from preserved to completely altered striatal dopamine uptake [35].

This study has some limitations. First, only a single night PSG recording was used. Additional nights could help to better evaluate RWA. Second, the study population is limited, thus the lack of the correlation between RWA activity and nigro-striatal dopaminergic function may be due to the limited sample size. However, the present study has the strength of having used not only the most common [123I]FP-CIT-SPECT, but also the [18F]PE2I-PET, which is expected to have a higer spatial resolution. Third, only one automatic method was used and analyzed. The use of several algorithms in the same group of subjects may allow a more realistic comparison of their accuracy. However, previous validation studies show great agreement between those methods [36, 37], and the average sensitivity and specificity reported for all the compared algorithms was generally around 80%, supporting the thesis that the automated methods developed to date may represent supporting rather than stand-alone diagnostic tools.

## Conclusions

The results of the present study suggest that the automatic method for the quantification of REM sleep without atonia (RWA) developed by Frandsen et al. has a good accuracy in recognizing REM sleep behavioral disorder, even when applied to an independent sample of subjects. However, the confirmed evidence of the visual method's superiority found in this study seems to indicate that automatic methods cannot yet, at present, replace manual scoring. The extended use of automatic methods in clinical settings could still bring considerable advantages in speeding up RWA analysis with a standardized approach. This is particularly relevant considering that iRBD is a known manifestation of alpha-synucleinophaty in its prodromal stage. Early identification of individuals in neurodegeneration prodromal stages is nowadays a major challenge for clinicians and researchers. Standardized automatic techniques for the analysis of sleep muscle activity may be capable of reducing costs, diagnostic delays and operator-dependent differences, therefore representing an important resource in implementing large scale screening processes. Finally, in our study we also suggested that the entity of motor activity during REM sleep is not correlated with nigro-striatal dopaminergic function.
